# Genomic and Proteomic Analyses of *Salmonella enterica* Serovar Enteritidis Identifying Mechanisms of Induced *de novo* Tolerance to Ceftiofur

**DOI:** 10.3389/fmicb.2018.02123

**Published:** 2018-09-10

**Authors:** Devon Radford, Philip Strange, Dion Lepp, Marta Hernandez, Muhammad Attiq Rehman, Moussa Sory Diarra, S. Balamurugan

**Affiliations:** Guelph Research and Development Centre, Agriculture and Agri-Food Canada, Guelph, ON, Canada

**Keywords:** *Salmonella enterica* serovar Enteritidis, antibiotic resistance, ceftiofur, β-lactam, *de novo* mechanisms

## Abstract

With the alarming proliferation of antibiotic resistance, it is important to understand the *de novo* development of bacterial adaptation to antibiotics in formerly susceptible lineages, in the absence of external genetic input from existing resistance pools. A strain of ceftiofur susceptible *Salmonella enterica* serovar Enteritidis ABB07-SB3071 (MIC = 1.0 μg/ml) was successively exposed to sub-MIC of ceftiofur to allow its adaptation for tolerance to a concentration of 2.0 μg/ml of this antibiotic. Genomic and proteomic comparative analyses of the parental strain and induced tolerant derived lineages were performed to characterize underlying mechanisms of *de novo* adaptation (tolerance). Expression and localization of specific drug-, heme-, sugar-, amino acid-, and sulfate-transporters were altered, as was the localization of the cell membrane stabilizing protein OsmY in the tolerant strains adapted to 2.0 μg/ml compared to the parental isolate lines. This redistribution of existing transporters acts to minimize the concentrations of ceftiofur in the periplasm, by decreasing facilitated import and increasing active efflux and cytosolic sequestration as determined by high performance liquid chromatography quantification of residual total and extracellular ceftiofur after growth. Genetic, subcellular localization, and abundance changes of specific regulators of transcription, translation, and post-translational dynamics in the derived ceftiofur tolerant lineages decrease metabolic strain on cell walls and enhance periplasmic envelop stability against stress. This produces slower growing, more tolerant populations, which deplete free ceftiofur concentrations significantly more than susceptible parental populations (*P* < 0.05), as measured by recoverable levels of ceftiofur from cultures of equivalent cellular density incubated with equal ceftiofur concentrations. Genetic and abundance changes to specific carbon and nitrogen metabolism enzymes, not traditionally associated with beta-lactam metabolism, establish an enzymatic framework with the potential to detoxify/degrade ceftiofur, while mutations and changes in subcellular localization in specific cell surface factors enhance the stability of the Gram-negative cell envelop despite the compromising effect of ceftiofur. The observed changes highlight generalizable mechanisms of *de novo* tolerance without horizontal gene transfer, and thus can inform policies to combat antibiotic tolerance and minimize induction of *de novo* tolerance.

## Introduction

*Salmonella* spp. infections are among the top three most prevalent sources of food-borne illness in Canada causing over 87,000 illness per year, and are an ongoing global health concern (Varga et al., 2015). *Salmonella* causes severe illness, economic losses, and potentially death in at risk groups, with the serovar Enteritidis being a major culprit with increasing prevalence in recent decades (Diarra et al., 2014; Varga et al., 2015). As zoonotic pathogens, *Salmonella* spp. impacts both human health and agriculture making its biocontrol of interest to both sectors. Yet with the proliferation of antibiotic resistance in both sectors the need to understand how this pathogen changes and adapts to evade control strategies is a pressing need. As cephalosporins are among the front line antibiotics for the treatment of salmonellosis in humans the increasing prevalence of extended-spectrum cephalosporin resistant *Salmonella* in North America and Europe is particularly concerning (Liakopoulos et al., 2016).

Closely following the discovery and human application of antibiotics came the discovery of antibiotic resistance (Sauvage et al., 2008), and mechanistic questions of how bacteria change from being inhibited by a particular antibiotic to gaining tolerance allowing growth (Aminov, 2010). Phylogenetic and archeological metagenomics studies have traced the origins of antimicrobial resistance genes into prehistory, millennia before the modern “antibiotic era” (Aminov, 2010). Thus antimicrobial resistance acquisition processes are innate and ancient but may be exacerbated through the widespread use of antibiotics, especially in the absence of clear understandings of how tolerance develops. Resistance describes the inherited ability to grow at relatively high concentrations of a substance (Brauner et al., 2016), whereas a tolerant organism is heritably able to grow at higher levels of a substance than an ancestor, but may or may not be a high enough level to qualify as resistance.

Five general modes of acquired tolerance have been proposed; structural modification of antibiotic targets to minimize or abolish interaction, production of drug binding proteins to sequester drugs away from targets, increased expression of drug efflux pumps to minimize the intracellular concentration to tolerable levels, insulation of cells in drug impermeable biofilms and capsules, and enzymatic detoxification of antibiotics (Sauvage et al., 2008; Aminov, 2010; Jones and Howe, 2014). Characterizations of the genetic and proteomic processes involved in developing tolerance have been largely theoretical based on observations after the fact. While horizontal gene transfer accelerates the spread of specific antibiotic resistance genes, the processes underlying the origins of tolerance in the absence of pre-existing resistance determinants remain under-characterized.

β-Lactam antibiotics, such as penicillin, cephalosporin including ceftiofur, traditionally function by irreversibly binding the conserved active site in the C-terminal transpeptidase domain of PBPs blocking cross-linking between peptidoglycan peptide subunits (Sauvage et al., 2008). This binding occurs through the amide of the conserved β-lactam ring which gives the group its name. A major mode of resistance to β-lactams in *Enterobacteriaceae* has been the expression of enzymes, termed β-lactamases, which cleave and decarboxylate the conserved 4-carbon β-lactam ring thus preventing binding (Sauvage et al., 2008; Liakopoulos et al., 2016). Tolerance is also observed from over-production of functional and bait PBPs, and/or mutations to the binding site to reduce drug affinity (Sauvage et al., 2008), through decreased permeability of the cell envelope to antibiotic, and increased drug efflux through active transport (Medeiros et al., 1987; Nikaido, 2009). β-Lactamases are particularly advantageous for resistance due to the essential multifunctional nature of PBPs limiting the tolerance for mutation (Sauvage et al., 2008). These enzymes act as D-alanine carboxy-peptidases, peptidoglycan transpeptidases, glycan transglycosylases, and peptidoglycan endopeptidases essential to the maturation and stability of cell walls in both Gram-positive and Gram-negative bacteria (Sauvage et al., 2008). Latter generation β-lactam antibiotics, such as ceftiofur, conserve the β-lactam ring along with iminomethoxy/ketoxime groups (R-N-O-CH_3_) structural contexts not cleavable by known β-lactamases, yet efficient in binding diverse PBPs (Vilos et al., 2012) enhanced by the presence of an amino thiazole ring. With insensitivity to β-lactamases, variable resistance to ceftiofur-related antibiotics has been observed across diverse and related pathogenic species, with some strains showing strong β-lactamase independent tolerance through other mechanisms (Diarra et al., 2014). Mechanisms of such tolerances and how they might arise within formally susceptible lineages remain incompletely characterized.

In this study, we induced stable tolerance to ceftiofur in lineages descended from a susceptible *Salmonella enterica* serovar Enteritidis strain ABB07-SB3071 isolated from chicken and investigated the underlying mechanisms of adaptation to this antibiotic using proteomic and genomic analysis. This provides novel insight into the systematic effects of induced stable ceftiofur tolerance in the absence of horizontal transfer of genetic information from a pre-existing resistant population.

## Materials and Methods

### Growth and Passaging of *Salmonella* for Induced Ceftiofur Tolerance

A ceftiofur susceptible (minimal inhibitory concentration: MIC = 1.0 μg/ml) strain of *Salmonella* Enteritidis ABB07-SB3071 (isolate 3346) from our collection was used (Diarra et al., 2014) in this study to induce ceftiofur tolerance. Based on the ceftiofur MIC against the ABB07-SB3071 strain, six single colonies of this bacterium were inoculated and grown in MHB at 37°C, 100–150 rpm shaking with sub-MIC of ceftiofur (0.5 μg/ml). Every 48 h cells were transferred (500 μl to 50 ml) to fresh MHB with 0.5 μg/ml ceftiofur until an OD of 1.0 was achieved within 48 h. Then the concentration was increased to 0.75 μg/ml and the above step repeated every 48 h until an OD of 1.0 was achieved within 48 h. Concentration of ceftiofur was increased by 0.25 μg/ml increments and cells grown by repeated transfer every 48 h until they reach an OD of 1.0 within 48 h. This was repeated until *Salmonella* Enteritidis became tolerant to 2.0 μg/ml. Thus six populations were isolated from the susceptible initial, 1.0 and 2.0 μg/ml tolerant stages of the passaging process, for a total of 18 isolates.

After at least 15 passages (about 1300–2100 generations), growths of the resulting lineages were examined in the presence of ceftiofur at concentrations between 0.0 and 2.0 μg/ml in MHB using a PowerWave XS spectrophotometer to measure OD_600_ after 48 h. Turbidity was adjusted against 0.5 McFarland Standard for a starting concentration of ∼5 × 10^5^ CFU/ml in 200 μl of MHB with ceftiofur.

Lineages with tolerance up to 2.0 μg/ml were then passaged using the same criteria in ceftiofur-free Müller–Hinton II agar (MHA) to examine the stability of induced ceftiofur tolerance in the absence of selective pressure. Genetic stability of the *de novo* increase in tolerance was evaluated based on growth on MHA containing 2.0 μg/ml ceftiofur after one to three passages in ceftiofur-free media to determine if the changes were heritable or lost without active selection to maintain them.

To quantify changes in antimicrobial susceptibility, the MIC values of several other antimicrobial agents were determined against the parental susceptible strain and its ceftiofur tolerant-derived lineages, using the Sensititre broth microdilution automated system (Thermo Scientific^TM^, Mississauga, ON, Canada) according to the Clinical and Laboratory Standard Institutes recommendation (Clinical and Laboratory Standards Institute [CLSI], 2018).

### Comparative Analysis of Protein Abundances Between Differentially Resistant *Salmonella* Enteritidis ABB07-SB3071 Lines by 2D-DIGE

#### Separation of Dye-Labeled Soluble Proteins by Size and Isoelectric Point by 2D-DIGE

The studied susceptible *Salmonella* Enteritidis isolate and its derived ceftiofur tolerant lineages were grown in MHB containing 0.0, 1.0, or 2.0 μg/ml ceftiofur, to an OD_600_ of 1.0. Bacterial cells were pelleted with 10,000 × *g* of centrifugation for 15 min, resuspended in 1.0 ml of lysis buffer (8.0 M urea, 30 mM Tris, 4% CHAPS, 10 μl phenylmethane sulfonyl fluoride), and mechanical shearing lysed with a 0.5-mm BioBead bead beater (Mo Bio PowerLzyer24), oscillating at 2500 rpm for 1 min, repeated five times with 1 min rests on ice between cycles. Lysates were treated for 15 min at room temperature with DNase/RNase mix (GE Healthcare Life Sciences) to degrade nucleotide contamination. Protein was precipitated at -20°C in acetone overnight, dried to remove residual acetone, and then resuspended in 1.0 ml of lysis buffer.

Protein samples were quantified, normalized, and labeled according to manufacture specifications (GE Healthcare Life Sciences, 2-D Quant Kit) and established methods (Beckett, 2012). Gels were also prepared according to these methods. The pH of the purified protein samples was adjusted to 8.5 by titration prior to labeling. Dye labeling was performed on ice using 4.0 μl of dye stock (1.0 nM of dye/μl of DMF) for every 100 μg of total protein, blocking with 10 mM L-lysine-free base (Beckett, 2012).

Total soluble protein from each treatment was purified in four technical replicates, split into paired samples then labeled with either Cy2 or Cy5. Pooled control samples were prepared containing 4.16 μg of total protein from each of the four replicates of the 0.0, 1.0, and 2.0 μg/ml treatments for a total of 50 μg of protein per standard, and then labeled with Cy3. Each gel was loaded with 50 μg of Cy3, Cy2, and Cy5 labeled samples and run in one dimension on a pH gradient from 4.0 to 7.0 for separation by isoelectric points, then transferred and run in the second dimension on a 12% SDS–PAGE for separation by size. Complementary Cy2 and Cy5 dye swap samples were run to detect differential dye binding artifacts. All six DIGE gels were imaged using a BioRad ChemiDoc MP for the established excitation and emission spectra of Cy2, Cy3, and Cy5.

### Computational Analysis of Protein Abundance

ImageMaster 2D Platinum software (GE Healthcare Life Sciences) was used to analyze relative protein abundances between parental and adapted lineages. Digitized images of the six 2D-DIGE gels were organized as three matched hierarchical sets of two dye-swapped gels, with three dye exposures per gel, were loaded into the software for a total of 18 images. Four landmark protein spots were chosen for their conservation across all 18 images, focusing on definite but not over exposed conserved spots. The estimated molecular weight distribution within gels was defined based on manual annotation of Thermo PrecisionPlus Kaleidoscope dye-labeled protein ladder run in parallel with the size dimension of the protein samples. The estimated pI distribution was defined with the left and right bounds of gels as pH 4.0 and 7.0.

Matchable protein spots within DIGE image sets for the same gel were automatically matched by the validated ImageMaster algorithms. Artifact spots from gel bounds and the ladder were manually removed. Matchable spots between gels were then automatically determined using the ImageMaster algorithms. Manual curation by eye was used to resolve ambiguous matchings to account for the confidence limitations of automated matching, which requires higher conformity between gels than necessary for by-eye spot matching.

Quantification and normalization statistics were extracted from these matched gel systems and imported into Microsoft Excel to identify changes in relative specific protein abundances between treatments. Spot value, also known as volume ratio, was used as metric for comparison of protein spots between treatments. This was calculated as (volume of a treatment spot)/(volume of the matching Cy3 control spot), normalized assuming the overall volume ratio for all spots in two images should have a ratio of one. Mean spot value, and mean normalized spot value [(spot value-central tendency)/dispersion], within treatments was calculated for each matched spot. Mean spot values, and mean normalized spot values, were compared between treatments to identify spots which differ in value more than twofold. Mean spot values, and/or mean normalized spot values, differing more than twofold between treatments were evaluated for statistical significance of spot-wise differences between treatments using Welch’s two-sample *T*-test for samples of unequal variance. To correct for multiple hypothesis testing a Bonferroni-corrected *P*-value cut-off for an error rate of 0.05 was used (Dunn, 1961). Descriptive statistics were extracted for spots differing by more than twofold between treatments and significantly different based on the *T*-tests. Preliminary protein spot identities were predicted based on estimated pI and molecular weight compared to the compiled proteome of sequenced annotated *S. enterica* subsp. *enterica* serovar Enteritidis strains from NCBI (BioProjects: PRJEA30687, PRJNA219482, PRJNA244356, PRJNA273513, and PRJNA284328).

### Identification of Differentially Enriched Proteins by Mass Spectrometry

Protein spots found to differ significantly in abundance between susceptible and tolerant lineages by DIGE were matched by eye to a Coomassie blue stained 2D-PAGE and excised with a clean scalpel. Before excision the gel was rinsed three times in Milli-Q water with shaking for 5 min to remove unbound soluble contaminants. A band of gel without evident protein was excised as a negative control for background protein contamination. Each gel sample was minced into approximately 1.0 mm^2^ pieces, and then placed in individual 0.65 ml siliconized tubes (VWR). Three 10 min washes with 100 μl of 25 mM NH_4_HCO_3_ in 50% acetonitrile were used to remove the Coomassie stain from the gel fragments. Destained gel samples were treated with 100 μl aliquots of 100% acetonitrile until the gel fragments became white and shrunken. Thirty minutes incubation in 100 μl of DTT in 50 mM NH_4_HCO_3_ converted the proteins to a reduced state. Samples were reshrunk in 100% acetonitrile, followed by alkylation with 100 μl 55 mM iodoacetamide (30 min at room temperature in dark). Samples were washed in 200 μl of 50 mM NH_4_HCO_3_ for 15 min, then reshrunk in 100% acetonitrile and dried by SpeedVac for 20 min. After drying, 10 μl of 11.1 μg/ml trypsin (Sigma product No.: T6567) in 0.06 mM HCl, 50 mM NH_4_HCO_3_ solution was added to each sample, and allowed to rehydrate and digest for 1 h at room temperature. After rehydration an additional 50 μl of 50 mM NH_4_HCO_3_ solution was added to each sample and incubated at 37°C for 16–18 h.

After digestion samples were briefly vortexed and centrifuged, 50 μl of water was added to each sample, followed by 2 min vortexing and brief centrifuge. 10.0-min bath sonication followed by brief vortex and 30.0 s centrifuge served to solubilize the peptides out of the gel fragments into solution. This supernatant (containing tryptic peptides) was transferred into new tubes. Two rounds of further peptide extraction were formed adding 75 μl of 5% formic acid in 50% acetonitrile was added to the gel pellet in the first tube, with 2 min vortexing, followed by centrifugation, and 5 min sonication, only sonicating the first round of extraction. The resulting supernatants were removed and combined with the earlier peptide containing supernatant. This combined supernatant was dried to 10–15 μl using a SpeedVac, then cleaned with C18 ZipTips (Millipore). Purified protein samples were sent to the University of Guelph, Advanced Analytics Center for mass spectrometry peptide fingerprinting by matrix-assisted laser desorption/ionization time of flight (MALDI-ToF).

#### HPLC Analysis of Ceftiofur Stability in the Susceptible Parental Strain and Derived Tolerant Daughter Lineages

**FIGURE 1:**
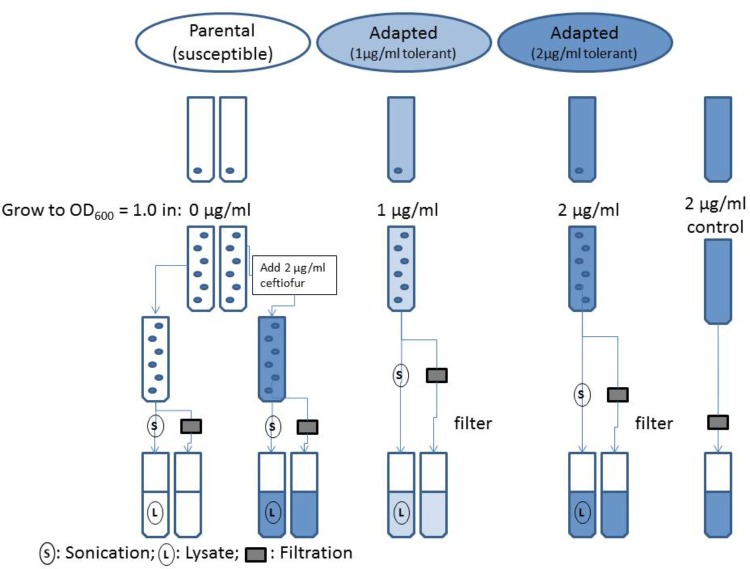
Flowchart of evaluation of ceftiofur localization in ceftiofur susceptible and tolerant cultures.

Isolates of the susceptible parental strain and adapted ceftiofur tolerant lineages of *Salmonella* Enteritidis were grown to OD600 = 1.0 in MHB (pH 7.2), with 0.0, 1.0, and 2.0 μg/ml ceftiofur respective to the established levels of tolerance for the ceftiofur susceptible and tolerant lines (**Figure 1**). A sterile tube of MHB with 2.0 μg/ml ceftiofur was incubated in parallel with the adapted strain. After growth the samples were split into two parallel analysis streams to compare the extracellular ceftiofur concentration and total ceftiofur concentration inside and outside the cells. The cell suspension samples used for total ceftiofur quantification by HPLC were sonicated for a total of 2 min on ice alternating 10 s on, 10 s off over the course of 4 min, to release internal ceftiofur. Both sets of samples were then filtered sterilized to remove bacterial cells and large debris. The “extracellular” ceftiofur sample thus excludes the ceftiofur from within the unlysed cells, because these cells are filtered out along with any internal ceftiofur. The susceptible parental strain extracellular media and lysates were split into negative control samples with 0.0 μg/ml ceftiofur and positive control samples to which stock ceftiofur was added to a concentration of 2.0 μg/ml. Samples were mixed with 4.0 g/l tetrabutyl ammonium bromide acetonitrile buffer in a 30:70 sample to acetonitrile ratio. Samples were run as 10 μl injections on a Waters Spherisorb ODS2C_18_ HPLC column (150 × 4.6 mm, 5 μm, 80 Å) at a flow rate of 1.0 ml/min for 10 min with a mobile phase of 60% 3.5 g/l disodium hydrogen phosphate buffer (pH 5.5), 40% 4.0 g/l tetrabutyl ammonium bromide acetonitrile solution by volume based on established methods (Palur et al., 2013). Non-acetonitrile solutions were filter through 0.2 μm pore cellulose acetate filters (Sigma–Aldrich) for sterility and elimination of large particulates. Acetonitrile solutions were filtered through 0.45 μm filter paper resistant to the solvent to exclude insoluble particulates. Elution peaks were measured at 292 nm using an ultra-violet spectrophotometric detector, and quantified using Agilent OpenLAB software to produce a standard curve relating ceftiofur concentration to elution peak area.

### Whole-Genome Sequence Analysis

The curated genome sequence from *Salmonella* Enteritidis ABB07-SB3071 (BioProject: PRJNA273513, BioSample: SAMN03293343) was used as the reference dataset to define novel genomic changes relative to the derived lines tolerant to 2.0 μg/ml ceftiofur. To minimize cost and focus on the mutations causing the stronger shift toward ceftiofur tolerance, only the 2.0 μg/ml ceftiofur tolerant populations were sequenced. The non-redundant identifiers for these genes were extracted from the NCBI nucleotide database draft genome assembly of this *Salmonella* Enteritidis isolate (NZ_LAOU01000001-34).

Genomic DNA from parental and the adapted ceftiofur tolerant lineages of *Salmonella* Enteritidis was extracted and libraries were prepared using the Nextera XT kit (Illumina) according to the manufacturer’s instructions. Libraries were sequenced with a MiSeq instrument (Illumina) using the 600 bp v3 kit (Illumina) as previously described (Rehman et al., 2017). Sequencing reads were aggregated and analyzed for quality using in house adapted shell scripts based on variant calling using SAMtools (Vaughn, 2013) and the Tablet platform for visualization (Milne et al., 2013). A coverage cut-off of >10 and a quality cut off of >30 confident reads for each predicted polymorphism were used to exclude sequencing artifact noise. Predicted polymorphisms were compared across three set of pooled lineage pairs tolerant to greater than 2.0 μg/ml ceftiofur to identify conserved targets of tolerance-associated modification.

Physical structures were predicted by Phyre2 (Kelley et al., 2015) for proteins with conserved polymorphisms in the coding sequences. Where supported by confident models, functional effects were predicted based on localizations of polymorphisms within these predicted structures using Phyre Investigator (Kelley et al., 2015) and Swiss PDB viewer (Guex and Peitsch, 1997). Kompetitive allele-specific PCR (KASP) and targeted sequencing assays were performed but revealed no change at typing loci.

## Results and Discussion

### Repeated Passage on Sub-MICs of Ceftiofur Induces *de novo* Tolerance

Ceftiofur susceptible *Salmonella* Enteritidis ABB07-SB3071 was used to examine the development of *de novo* tolerance to ceftiofur. Successive and prolonged exposure of the susceptible isolate to ceftiofur concentrations between 0.5 and 2.0 μg/ml yielded lines with enhanced tolerances up to 2.0 μg/ml compared to the non-exposed parental isolate. These derived lineages with enhanced ceftiofur tolerance retained their enhanced tolerance even without continued selection, in the absence of ceftiofur for several generations. Based on Sensititre broth microdilution automated system results (Clinical and Laboratory Standards Institute [CLSI], 2018), MICs of ceftiofur and ceftriaxone, a closely ceftiofur-related antibiotic used in human medicine against the adapted lineages were 8.0 and 0.5 μg/ml compared to the parental strain (1.0 and 0.25 μg/ml). Compared to the parental strain, the 2.0 μg/ml ceftiofur-adapted lineages showed elevated MICs for several other antimicrobial agents including amoxicillin/clavulanic acid (2.0 vs. 8.0 μg/ml), ampicillin (1.0 vs. 16 μg/ml), chloramphenicol (8.0 vs. 16 μg/ml), ciprofloxacin (0.015 vs. 0.06 μg/ml), and nalidixic acid (2.0 vs. 8.0 μg/ml) (**Table 1**). These results clearly indicate that exposure of susceptible Enteritidis isolates to sub-MICs can lead to cross-resistance to multi-antimicrobials.

**Table 1:** Minimum inhibitory concentrations of ceftiofur tolerant lineages and the susceptible parental strain by Sensititre broth microdilution automated system.

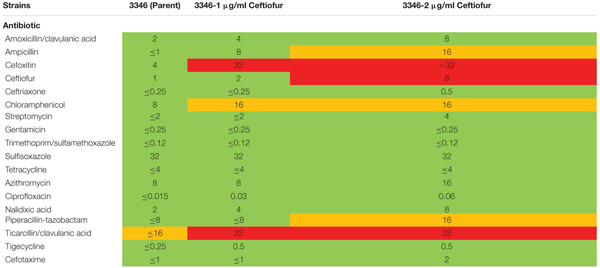

*Mean of biological replicates in each category. Red color, resistant; yellow color, intermediate; green color, susceptible.*

### Differential Susceptibility to Ceftiofur Associated With Distinct Changes in Abundance of Specific Proteins

Individual protein abundances were compared between the susceptible parental strain, and its derivative lineages able to grow in 1.0 and 2.0 μg/ml of ceftiofur to detect correlated changes in proteins contributing to tolerance. A Bonferroni corrected (Dunn, 1961) *P*-value cut-off of 0.00002659 was used to evaluate significance for the multiple hypothesis testing effects of the 1880 tests considered. Fifty-eight protein spots showed statistically significant differences in mean abundance greater than twofold between the samples of the susceptible parental strain and tolerant populations at the 1.0 and/or 2.0 μg/ml. Of these, 32 protein spots yielded meaningful predictions of protein identity by mass spectrometry fingerprinting (**Table 2**). Six of these spots contained more than one protein, defining a set of 38 proteins implicated in conferring the observed change in ceftiofur susceptibility. None of these 38 proteins are PBP homologs, nor are they β-lactamase homologs, the two protein families traditionally associated with acquired tolerance to ceftiofur-like antibiotics. The levels of these proteins showed difference between the three categories, consistent with the differences in tolerance and susceptibility.

**Table 2:** Significantly differentially abundant proteins between ceftiofur tolerant and susceptible lineages.

Average MW (Da)	Average pI (pH)	Description	Accession (gi)	Mass Spec Conf (-10logP)	Spot value fold difference	Response to ceftiofur
84678.17	5.41	Pyruvate dehydrogenase	WP_058222735.1	234.8	1 μg/0 μg: 2.34	Up
			KSU35937.1		2 μg/0 μg: 2.60	
64634	5.72	Predicted MFS transporter	AHS49296.1	25.4	1 μg/0 μg: 2.36	Up
					2 μg/0 μg: 2.51	
55549.28	5.28	Phase-1 flagellin	AAA53492.1	234.47	1 μg/0 μg: -6.40	Down
			AAA53494.1		2 μg/0 μg: -5.50	
55465.39	5.21	Trigger factor	WP_058107428.1	201.07	1 μg/0 μg: -5.70	Down
			WP_060629093.1		2 μg/0 μg: -4.93	
41725.08	5.20	GTP-binding protein YchF	WP_058115804.1	201.45	1 μg/0 μg: 2.42	Up
					2 μg/0 μg: 2.46	
41541.72	5.28	Phosphoglycerate kinase	WP_058818744.1	132.72	1 μg/0 μg: 2.52	Up
			WP_058812842.1		2 μg/0 μg: 2.11	
			WP_058116115.1			
40326 (runs as 37533.06)	6.59	MalE, maltose ABC transporter	WP_060453748.1	145.46	1 μg/0 μg: -7.49	Down
			KWR42205.1		2 μg/0 μg: -6.72	
39172 (runs as 37533.06)	6.59	Fructose-bisphosphate aldolase	WP_058214667.1	226.45		
			KSU42677.1			
			AAX66916.1			
41779.4 (runs as 38246.33)	6.05	Mannose-6-phosphate isomerase	WP_050954112.1	130.5	1 μg/0 μg: -4.80	Down
			GAS09170.1			
			CQJ97592.1		2 μg/0 μg: -3.80	
			WP_058821307.1			
			WP_060453903.1			
			WP_050957484.1			
			WP_058652129.1			
39759.5 (runs as 38246.33)	6.05	Glycerolphosphoryl-diesterphosphor- diesterase	WP_049264243.1	195.26		
37673.72	4.98	PTS fructose transporter, IIA subunit	WP_000487282.1	231.46	1 μg/0 μg: 3.65	Up
			CAR38077.1		2 μg/0 ug: 6.49	
			CAR33784.1			
37509.39	6.29	MalE-like maltose ABC transporter	WP_051575368.1	255.12	1 μg/0 μg: -6.82	Down
					2 μg/0 μg: -6.02	
37302.83	5.24	Bifunctional PTS fructose transporter, IIA/HPr subunit	GAR51273.1	208.39	1 μg/0 μg: 2.82	Up
			WP_000487280.1		2 μg/0 μg: 2.14	
			WP_058800431.1			
			WP_058671296.1			
36898.28	5.17	PTS fructose transporter, IIA/HPr subunit	WP_000487282.1	277.45	1 μg/0 μg: 3.59	Up
			WP_060569325.1		2 μg/0 μg: 4.12	
			WP_059347050.1			
35543 (runs as 35800)	5.90	L-Asparaginase II	WP_000394183.1	243.17	1 μg/0 μg: -4.51	Down
			EJH90481.1			
			EJH91134.1		2 μg/0 μg: -3.18	
40362.5 (runs as 35800)	5.90	Glycerophospho-diester phosphodiesterase, precursor	GAS41321.1	167.71		
			WP_058801313.1			
			GAR15821.1			
			GAR62889.1			
			KTZ36784.1			
34448.11	5.86	L-Asparaginase 2	WP_057516253.1	166.14	1 μg/0 μg: 5.09	Up
					2 μg/0 μg: 2.59	
31880.06	5.53	Elongation factor, Ts	WP_000808106.1	206.91	1 μg/0 μg: -5.46	Down
			WP_050959197.1		2 μg/0 μg: -3.63	
			ACN44422.1			
			ESF26854.1			
			AAX64123.1			
			WP_000808107.1			
			WP_000808108.1			
36561.45 (runs as 31438.5)	6.30	LsrB, autoinducer 2-binding protein, precursor	WP_060614895.1	196.08	1 μg/0 μg: -5.66	Down
			WP_000090738.1			
			WP_000090742.1		2 μg/0 μg: -4.37	
			WP_052971164.1			
			WP_058656188.1			
			WP_000090739.1			
			WP_000090734.1			
			WP_058651988.1			
			WP_024154537.1			
			WP_050189314.1			
			WP_058806004.1			
31292.5 (runs as 31438.5)	6.30	DapA, 4-hydroxy-tetrahydro- dipicolinate synthase	WP_046595969.1	147.54		
			WP_052901724.1			
			WP_058653368.1			
			WP_058673748.1			
			WP_050188264.1			
			WP_058109262.1			
			WP_000494019.1			
31426.22	6.65	LsrF, autoinducer 2 aldolase	WP_000774146.1	148.08	1 μg/0 μg: -5.05	Down
			WP_058649945.1			
			WP_024155380.1		2 μg/0 μg: -3.81	
			KSU44327.1			
			WP_046595658.1			
			WP_000774149.1			
35338.2 (runs as 31077.83)	5.74	Acetyl-CoA carboxylase carboxyltransferasesubunit alpha	WP_017441520.1	231.58	1 μg/0 μg: -4.92	Down
			WP_000055753.1			
			WP_020842713.1		2 μg/0 μg: -5.22	
			WP_000055752.1			
31600.36 (runs as 31077.83)	5.74	Pfk1, 1-phosphofructo-kinase	WP_053299797.1	79		
			WP_052928962.1			
			WP_058113434.1			
			WP_057514294.1			
			WP_050308994.1			
			WP_052936223.1			
29017.28	5.16	Predicted glycine/sarcosine/betaine reductase, ABC subunit	WP_060790288.1	155.49	1 μg/0 μg: 2.35	Up
			WP_002837524.1		2 μg/0 μg: 2.20	
			WP_004269617.1			
			WP_002835445.1			
			WP_002841334.1			
26844.72	5.93	Histidine ABC transporter substrate- binding protein HisJ	WP_001540524.1	172.07	1 μg/0 μg: -4.91	Down
			WP_058656046.1		2 μg/0 μg: -4.31	
23236.78	6.75	Arginine ABC transporter substrate- binding protein	WP_052936247.1	144.45	1 μg/0 μg: -4.67	Down
			KMT72690.1		2 μg/0 μg: -4.83	
			WP_000756586.1			
			WP_050195704.1			
			WP_050950073.1			
			WP_057516609.1			
			AAX64788.1			
22945.67	3.43	Glutamine-binding periplasmic ABC transporter substrate-binding protein	WP_046596508.1	168.62	1 μg/0 μg: -6.23	Down
			WP_000838672.1		2 μg/0 μg: -4.35	
22860 (runs as 22041.78)	5.44	Ribose 5-phosphate isomerase A	WP_024192175.1	128.61	1 μg/0 μg: -4.77	Down
			WP_024191163.1		2 μg/0 μg: -3.19	
			WP_046597656.1			
			CSP70625.1			
			WP_054192314.1			
			EFJ59281.1			
21108.82 (runs as 22041.78)	5.44	Elongation factor P	WP_050184945.1	89.85		
			WP_057483777.1			
			AAX68119.1			
19280.22	5.38	BON domain, periplasmic, osmotically inducible protein, OsmY	WP_049883528.1	149.98	1 μg/0 μg: -7.53	Down
					2 μg/0 μg: -6.66	
17937.06	5.40	RNA polymerase-binding transcription factor, DksA	WP_059295986.1	145.03	1 μg/0 μg: -5.53	Down
					2 μg/0 μg: -3.48	
17171.72	5.41	2-Cys peroxiredoxin/peroxidase	WP_001710966.1	170.03	1 μg/0 μg: -9.82	Down
			WP_031625066.1		2 μg/0 μg: -2.75	
			WP_023231242.1			
			WP_050963461.1			
			CNU06456.1			
15567	6.10	Ferric uptake regulator-like transcriptional repressor	WP_050158487.1	108.3	1 μg/0 μg: -6.09	Down
			WP_058112899.1		2 μg/0 μg: -2.96	
			WP_057483836.1			
			WP_050155516.1			
			WP_050152520.1			
			WP_046598395.1			
14160.94	5.89	H-Ns-like transcriptional regulator	WP_050195838.1	145.35	1 μg/0 μg: -5.56	Down
			CIE52848.1		2 μg/0 μg: -3.15	
			WP_045716493.1			
12961.06	5.93	Molecular chaperone GroES	WP_024139196.1	111.74	1 μg/0 μg: -4.40	Down
			WP_000027827.1		2 μg/0 μg: -3.92	
			EGE36591.1			
			AAX68114.1			
			EMR50022.1			
12073.06	4.89	50S ribosomal protein L7/L12	WP_038394387.1	63.09	1 μg/0 μg: -6.23	Down
			WP_060588498.1		2 μg/0 μg: -3.30	
11778.89	5.45	L-PSP enamine/imine deaminase	WP_000047544.1	90.31	1 μg/0 μg: -4.99	Down
			GAL38445.1		2 μg/0 μg: -3.74	
			WP_023241011.1			
			WP_046595790.1			
14994.22	5.87	DNA-binding transcriptional	WP_053445746.1	178.1	1 μg/0 μg: -6.64	Down
		regulator, H-Ns	WP_050195838.1		2 μg/0 μg: -3.92	
			WP_050194492.1			
			WP_045716493.1			


Three PTS fructose transporter subunits and a predicted MFS transporter showed increased soluble abundance while ABC transporters of histidine, arginine, and glutamine showed decreased soluble abundance in the ceftiofur tolerant lineages. Increased production and membrane incorporation of transporters acting as active drug efflux pumps or periplasmic exclusion systems against ceftiofur, such as the PTS and ABC transporters, would promote tolerance, as would decreased production and incorporation of transporters facilitating entry of the antibiotic to the periplasm (Nikaido, 2009). These transporters have also been implicated, along with the RND transporter family, in cross resistance to multiple antimicrobials (Nikaido, 2009). Comparison to other distinct susceptible and tolerant strains of *S.* Enteritidis in our collection revealed a number of the variants of RND-1 found in our system are associated with tolerance, even though they are present in both the parental and tolerant lineages we worked with. If coupled with ceftiofur degrading enzymes within the cytosolic compartment, transport of ceftiofur from the periplasm into the cytosol could also enhance tolerance, as PBPs are exclusively active in the periplasm (Sauvage et al., 2008). The MFS transporter being a passive transporter (Nelson and Cox, 2005) likely facilitates ceftiofur entry, and is sequestered from the cell envelope during ceftiofur tolerance giving the apparent increased soluble abundance. Despite ceftiofur being structurally distinct from the amino acids and sugars canonically associated with these transporters, ceftiofur does include functional groups similar to histidine, arginine, and glutamine and fructose. Thus, if the substrate binding sites are lax enough, these transporters might transport ceftiofur or derivatives. Indirect effects of transporter abundance and distribution may also be involved through cell wall stabilization effects of some transporter cargo, such as the membrane stabilizing OsmY protein (Yeats and Bateman, 2003), which requires export from the cytosol into the periplasm. *In vitro* evidence supports a relationship between higher glucose levels and ceftiofur instability (Dolhan et al., 2014) such that transporter-mediated altered sugar localization could influence ceftiofur degradation *in vivo*.

The OsmY protein family are Gram-negative periplasmic or outer membrane stress tolerance proteins forming bonds between phospholipids on the inner and outer membrane (Yeats and Bateman, 2003). In the unbound state OsmY is soluble, while in the membrane bound state it fractionates with the membrane (Yeats and Bateman, 2003). Thus, the decrease in soluble abundance of OsmY observed in the ceftiofur tolerant lineages is consistent with increased stabilization activity through association with the cell membranes. We also observed differences in OsmY and similar proteins between unrelated tolerant and susceptible strains. The observed cross resistance to multiple antimicrobial agents could be due to outer membrane protein changes such as OsmY (Nikaido, 2009).

The depletion of elongation factors Ts and P, 50S ribosomal protein L7/L12, RNA polymerase-binding transcription factor DksA, Fur-like transcriptional repressor, two H-Ns-like transcriptional repressors, the molecular chaperones GroES, and trigger factor, and the increase in GTP-binding protein YchF abundance is consistent with a complex rebalancing of the transcriptome and proteome composition to enable enhanced ceftiofur tolerance (Teplyakov et al., 2003; Susin et al., 2006; Tjaden et al., 2006; Hoffmann et al., 2010; Vabulas et al., 2010; Furman et al., 2012; Mandava et al., 2012).

Genetic depletion of GroES produces slow growth and long undivided filamentous cells with 96% of cells showing aborted z-rings and irregular incomplete septa (Susin et al., 2006). The level of GroES depletion we observed slows cell cycle progression, approximately twofold for the 2.0 μg/ml tolerant lineages compared to the susceptible parental strain. Reducing the cell division rate enhances tolerance to ceftiofur cell wall damage by reducing the incidence of division induced cell shearing, while increasing the accumulation of unfolded protein as a side effect. The latter effect would be partially mitigated by the predicted increase in DnaK activity from DksA depletion (Vabulas et al., 2010).

LsrB is the *Salmonella* receptor for the furanosyl borate diester, autoinducer 2 (AI-II), which is a quorum sensing signal (Miller et al., 2004). In the ceftiofur tolerant lines, the depletion of LsrB reduces sensitivity to AI-II and quorum sensing. The AI-II aldolase (LsrF) and seven other essential metabolic enzymes show decreased abundance in the ceftiofur tolerant lines: ribose 5-phosphate isomerase A, mannose-6-phosphate isomerase (MPI), 1-phosphofructokinase (Pfk1), fructose-bisphosphate aldolase (FBPa), glycerophosphoryl diesterphosphordiesterase, 4-hydroxy-tetrahydro-dipicolinate synthase (DapA), and acetyl-CoA carboxylase carboxyl transferase subunit-α. Depletion of DapA, MPI, Pfk1, acetyl-CoA carboxylase carboxyl transferase, FBPa, and glycerophosphoryl diesterphosphordiesterase alters cell wall biosynthesis dynamics to better tolerate the destabilizing effect of ceftiofur (Nelson and Cox, 2005).

2-Cys peroxiredoxin/peroxidase and L-PSP enamine/imine deaminase also showed decreased abundance in the ceftiofur tolerant lineages. L-PSP enamine/imine deaminase is involved in metabolizing atypical nitrogen sources (Lambrecht et al., 2012), while 2-Cys peroxiredoxin/peroxidase is involved in thiol-dependent oxidative stress response (Hall et al., 2009). Given the abundance of nitrogen and sulfur in ceftiofur, these enzymes may carryout off-target reactions with ceftiofur producing more toxic by-products, or may generate products which compete with ceftiofur for enzymes involved in antibiotic detoxification (Hall et al., 2009; Lambrecht et al., 2012).

**FIGURE 2:**
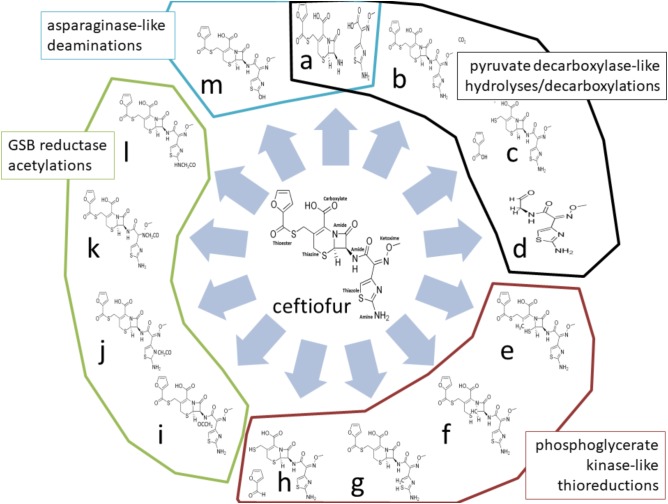
Theoretical ceftiofur degradation produces from interaction with pyruvate decarboxylase [**(a)** thioesterase hydrolysis; **(b)** beta-lactam decarboxylation; **(c)** amide hydrolysis; **(d)** multiple hydrolysis], phosphoglycerate kinase/reductase [**(e)** 1,6 thiazine reduction; **(f)** 1,2-thiazine reduction; **(g)** 1,5-thiazole reduction; **(h)** thioester reduction], glycine/sarcosine/betaine reductase [**(i)** secondary amide acetylation; **(j)** thiazole acetylation; **(k)** ketoxime acetylation; **(l)** amine acetylation], and asparaginase II [**(a)** amide hydrolysis; **(m)** amine hydrolysis].

Four enzymes showed greater than twofold increased abundance in the ceftiofur resistant lines: pyruvate dehydrogenase, phosphoglycerate kinase (PGK), L-asparaginase II, and a predicted glycine/sarcosine/betaine (GSB) reductase. Pyruvate dehydrogenases function through binding and decarboxylating electrophilic ketones (Ciszak et al., 2003), and are known to reduce the antibiotic metronidazole through a ketone independent reaction targeting the nitro group (Jones and Howe, 2014). These activities function through the thiazole ring of a bound thiamine pyrophosphate in the active site (Ciszak et al., 2003). Ceftiofur contains an aminated thiazole ring with similar conformation (**Figure 2**). Thus, ceftiofur or a derivative may be a target or competitor for this enzyme. The ceftiofur structure includes three electrophilic ketone-like groups; two amides (tertiary amide in β-lactam and secondary amide) and a thioester (**Figure 2**), which could be decarboxylated by this enzyme similar to how conventional β-lactamases function (Sauvage et al., 2008; **Figures 2a–d**). Hydrolytic cleavage of the thioester produces desfuroylceftiofur, the primary cited degradation product of ceftiofur in mammals, and 2-furoic acid which can act as an antimicrobial or serve as a carbon and energy source for bacterial metabolism through conversion to α-ketoglutarate (Li et al., 2011). Desfuroylceftiofur is as toxic as ceftiofur to Gram-negative bacteria, but more reactive forming conjugates with reduced antibacterial activity (Li et al., 2011). Further hydrolysis at the β-lactam ring of desfuroylceftiofur would generate the non-bactericidal products cef-aldehyde (**Figure 2d**), observed in waste water from farms using ceftiofur (Li et al., 2011), and1,3-thiazine-2-keto-4-carboxy-5-methyl-mercaptan (C_6_H_7_O_3_NS_2_). The 1,3-thiazine-2-keto-4-carboxy-5-methyl-mercaptan can be further degraded to homocysteine and feed into methionine and cysteine biosynthesis.

Phosphoglycerate kinase may contribute to detoxification of ceftiofur through thiol reduction similar to human PGK’s thiol reductase activity on plasmin in tumor suppression (Lay et al., 2000). There are two sulfides (thiazine and thiazole) and a thioester in unmodified ceftiofur (**Figure 2**). Reduction of any of the sulfides to thiol, or reductive cleavage of the thioester (**Figures 2e–h**), or reduction of the thiol generated by thioester cleavage (**Figure 2c**) would inactivate ceftiofur. Reductive cleavage of the thioester produces desfuroylceftiofur and 2-furfural. Oxidation of the thiazine has been observed *in vitro* (Lim et al., 2011). In mammals, the thioester bond is rapidly cleaved forming desfuroylceftiofur, which is metabolized to the disulfide dimer and amino acid conjugates followed by catabolism as needed (Dolhan et al., 2014), such that *Salmonella* may utilize analogous pathways for β-lactamase independent detoxification.

Glycine/sarcosine/betaine reductases catalyze the production of glycine, *N*-methylglycine, or *N*,*N*,*N*-trimethylglycine from acetyl phosphate and ammonia or methylated amines (Wagner et al., 1999). Ceftiofur includes a terminal primary amine structurally similar to sarcosine, two amides, a secondary ketoxime, and a thiazole as possible targets for acetylation (**Figures 2i–l**). Acetylation at any of these sites may be sufficient to prevent antibiotic activity, and feed into pathways analogous to desfuroylceftiofur–amino acid conjugate catabolism in mammals (Dolhan et al., 2014). Acetylation of one of these amides has been observed in the degradation of ceftiofur in swine tissues following cleavage of the thioester (Beconi-Barker et al., 1995). Modifications of the ketoxime group that exposed the β-lactam ring to attack would enable basally expressed β-lactamases to efficiently detoxify ceftiofur without increasing total levels of β-lactamase protein.

L-Asparaginase II proteins are high-affinity, constitutively periplasmic enzymes converting L-asparagine to L-aspartate and/or glutamine to glutamate as part of cell wall biosynthesis (Nelson and Cox, 2005). In the ceftiofur resistant lineages, this enzyme showed 2.59- to 5.09-fold increased abundance. Ceftiofur lacks the primary amide [R–(C=O)–NH_2_] conserved between asparagine and glutamine, but does include a terminal primary amine attached to a similarly electrophilic thiazole ring, along with its two internal amides as possible sites for cleavage or deamination by asparaginase (**Figures 2a,m**). Increased periplasmic asparaginase may also enhance production of glutamate-derived peptidoglycan to partially counter the anti-crosslinking effects of ceftiofur. Increased abundances of proteins with these enzymatic activities are consistent with the observed biotic depletion of free ceftiofur in cultures growing the resistant lineages, as detected by HPLC.

### Ceftiofur Tolerant *Salmonella* Enteritidis Lineages Deplete the Quantity of Free Ceftiofur

Under the HPLC conditions described in our methods, a distinct peak was observed in ceftiofur containing standards and samples occurring at an average retention time of 2.247 s (σ = 0.01255), which scales with ceftiofur concentration from 0.25 to 8.0 μg/ml remaining distinct from background as low as 0.25 μg/ml inclusive. Ceftiofur-free MHB includes a minor component with a partially overlapping peak centered at an average retention time of 2.257 s (σ = 0.008886), which was subtracted from ceftiofur peak areas to normalize for background signal. This background component, likely non-specific tryptophan containing tripeptides, is depleted during *Salmonella* Enteritidis growth, yielding a lower background signal in bacterial controls and samples as these compounds are converted to larger macromolecules. No significant abiotic degradation of ceftiofur signal over time was found in sterile MHB at 37°C over 48 h, the period needed for the ceftiofur tolerant *Salmonella* to fully grow (*T*-test *P*-value >0.3). This supports the stability of ceftiofur under these conditions without biodegradation, expanding on prior stability trials in saline (Dolhan et al., 2014).

**FIGURE 3:**
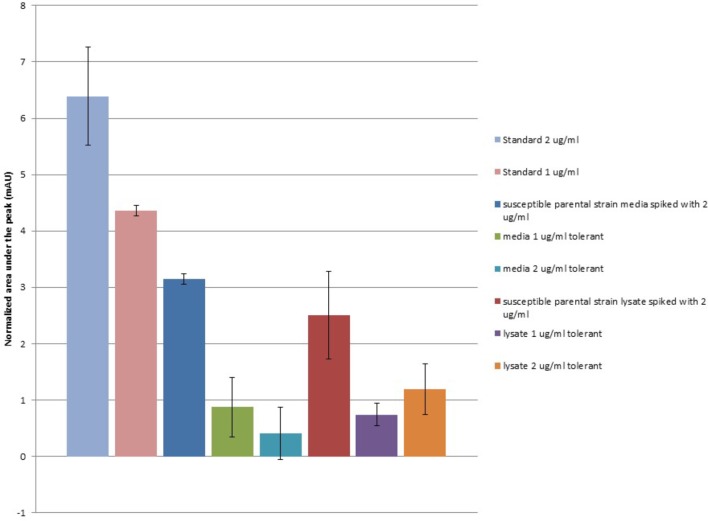
Ceftiofur retention in closely related ceftiofur susceptible and tolerant lineages of *Salmonella* Enteritidis. Normalized against background of elution spectra of ceftiofur-free MHB, susceptible parental *Salmonella* Enteritidis strain spent MHB, and sonicated susceptible parental *Salmonella* Enteritidis strain cell lysate in MHB as appropriate.

When extracellular media from 48 h growth of the ceftiofur susceptible parental *Salmonella* Enteritidis strain and its derivate lineages tolerant to 1.0 or 2.0 μg/ml of ceftiofur were examined, the levels of recoverable ceftiofur HPLC signal were significantly lower (*T*-test *P* = 0.003478) than the standards of the same concentrations from the control MHB (**Figure 3**). From an input concentration of 2.0 μg/ml interaction with the susceptible parental strain reduces the free ceftiofur signal in the media to 3.15 mAU, the equivalent of less than 1.0 μg/ml. This likely results from binding to antibiotic target proteins in latent cell wall debris (Vilos et al., 2012). Media from the 1.0 μg/ml ceftiofur tolerant culture showed a further 1.8-fold drop in ceftiofur signal, 0.873 mAU, from what would be expected based on the susceptible parental strain positive control, 1.575 mAU. The 2.0 μg/ml ceftiofur tolerant culture carried this further with a 7.8-fold lower than expected ceftiofur signal of 0.407 mAU (*T*-test *P* < 0.001). Strikingly after 48 h growth there was less free ceftiofur detectable in the 2.0 μg/ml ceftiofur tolerant cultures, which started with 2.0 μg/ml ceftiofur, than in the 1.0 μg/ml ceftiofur tolerant cultures which started with 1/2 the concentration. This supports some form of more extensive interaction (sequestration, degradation, or binding) between the tolerant lineages and ceftiofur compared to the susceptible parental strain. Cell densities did not vary substantially between cultures, suggesting these differences in free ceftiofur are not fully explained by binding to target proteins.

Samples of these cultures were mechanically lysed by sonication to release cytosolic ceftiofur to assess total unbound ceftiofur remaining in both the extracellularly and in the cytoplasm after resistant lineage growth. The level of free ceftiofur detectable in the positive control prepared from susceptible parental strain lysate again showed a significant drop in signal (*T*-test *P* < 0.005, **Figure 3**); lower on average than the signal from the extracellular samples but with more variability, suggesting sonication released more binding partners for ceftiofur. The total ceftiofur signals from the 2.0 μg/ml tolerant cultures were 2.9-fold higher than the levels observed from the extracellular media, suggesting tolerance in that lineage includes increased active internalization of ceftiofur in the cytoplasm sequestered from the drug target in the periplasm. The total ceftiofur signals from the 1.0 μg/ml tolerant cultures were lower but similar to the levels observed from the extracellular media (0.74 mAU vs. 0.873 mAU, *P* = 0.31), suggesting cytoplasmic sequestration is not as active a mode of tolerance at the lower concentration. In both cases, the levels of detectible ceftiofur were lower than expected from the susceptible parental strain samples (1.0 μg/ml: 59%, *P* = 0.066, 2.0 μg/ml 48%, *P* = 0.042), suggesting tolerance is accompanied or facilitated by increases in biochemical interaction between ceftiofur and these bacteria, which may include degradation or increased binding within the insoluble fraction in addition to the increase in cytosolic sequestration.

These results are consistent with some level of active enzymatic degradation of ceftiofur, but not sufficient to rule out other explanations such as increased insoluble sequestration. As expected peaks consistent with predicted ceftiofur degradation products were observed but were too similar in intensity and elution profile to the controls to confirm or refute if the missing ceftiofur was being converted as hypothesized. Future *in vitro* studies with purified enzymes may address these hypotheses biochemically.

### Differential Susceptibility to Ceftiofur Associated With Distinct Mutations in the *Salmonella* Enteritidis Genome

Comparison of the reference genome (BioProject: PRJNA273513, BioSample: SAMN03293343) to whole genome sequencing reads from the lineages with induced ceftiofur tolerance (>2.0 μg/ml) identified 27 loci with SNPs or indels specific to and conserved in all three samples from the ceftiofur tolerant lineages. There were also 15 other loci with SNPs or indels specific to and conserved in two out of three samples from the ceftiofur tolerant lineages (≥2.0 μg/ml). These polymorphic loci are listed in **Table 3**. None of these 43 genes are PBP homologs, nor are they annotated β-lactamase homologs, the two protein families traditionally associated with acquired tolerance to ceftiofur-like antibiotics (Sauvage et al., 2008; Liakopoulos et al., 2016). Seventeen genes show non-synonymous conserved changes within the coding sequence, while 27 showed changes to the upstream region potentially altering promoter, repressor, and enhancer activities, with five showing conserved polymorphisms in both the upstream and coding regions. Three showing only synonymous changes. Of these 43 genes cds200, cds201, cds1513, cds1514, cds2374, cds4043, cds4044, cds4045, and cds4151 were encoded at the edges of contigs preventing definitive sequence confirmation beyond the beginning or end of the contigs. To evaluate the impact of polymorphisms in these incomplete proteins, complete sequences were reconstructed based on complete ORFs with identical matching sequences from other *S. enterica* strains.

**Table 3:** Sites of genomic polymorphisms between ceftiofur tolerant and susceptible lineages.

Lines	SNP/indel site	Locus	Accession	Description
A,B,C	Promoter	cds1107	WP_001036949.1	Galactose ABC transporter substrate-binding protein
A,B,C	Promoter	cds1116	WP_000488245.1	Catecholate siderophore receptor, CirA
A,B,C	CDS	cds1513	WP_001238304.1	Hemelyase, subunit CcmH
A,B,C	Promo. & CDS	cds1514		Pathogenicity island 2 effector protein, SseI
A,B,C	CDS (syn)	cds1181	WP_001169931.1	Surface-exposed virulence protein BigA
A,B,C	Promoter	cds1942	WP_001156217.1	tRNA 2-thiocytidine(32) synthetase, TtcA
A,B,C	CDS (syn)	cds1943		Integrase
A,B,C	CDS	cds199	WP_001001154.1	Oxaloacetate decarboxylase, γ subunit
A,B,C	Promoter	cds200	WP_000150408.1	Oxaloacetate decarboxylase
A,B,C	CDS	cds201	WP_000490324.1	Uncharacterized outer membrane protein, YjiK
A,B,C	Promoter	cds2050	WP_000203802.1	Aromatic amino acid exporter
A,B,C	CDS	cds2051	WP_046333564.1	Sulfate ABC transporter substrate-binding protein
A,B,C	Promoter	cds2052	WP_000181597.1	Formate dehydrogenase-*N* subunit alpha
A,B,C	Promoter	cds2123	WP_000534697.1	Dimethyl sulfoxide reductase, subunit H
A,B,C	Promoter	cds2124		Dimethyl sulfoxide reductase, subunit H
A,B,C	CDS	cds2374	99.5% identical to AHQ19329.1	Predicted inner membrane, Ig-like domain repeat, molybdopterin-binding oxidase/adhesin
A,B,C	CDS	cds3091	WP_000888535.1	Heme exporter protein CcmA
A,B,C	Promoter	cds3092	WP_000990039.1	Heme exporter protein, CcmB
A,B,C	Promoter	cds3145, cds3146	WP_000602034.1	Membrane protein, ATP:dephospho-CoA triphosphoribosyl transferase, CitG
A,B,C	CDS	cds4043	AHR65859.2	Translation elongation factor, Tu
A,B,C	Promo. & CDS	cds4044	WP_000444887.1	Oxaloacetate decarboxylase, β-subunit
A,B,C	Promo. & CDS	cds4045	WP_000150436.1	Oxaloacetate decarboxylase, α-subunit
A,B,C	Promo. & CDS	cds4151	ANF21424.1	Thiol:disulfide interchange protein
A,B,C	Promoter	cds4152	WP_000135399.1	Helix-turn-helix transcriptional regulator
A,B,C	CDS (syn)	cds4153	WP_016740776.1	Translation elongation factor, Tu
A,B,C	Promoter	cds4400	WP_001145257.1	Integrase
A,B,C	Promoter	cds4402	WP_001224051.1	Transposase
A,C	Promoter	cds343	WP_001705876.1	Sugar translocase
A,C	Promoter	cds344	WP_000703599.1	Bactoprenolglucosyl transferase
A,C	Promoter	cds345	WP_000400616.1	Acyltransferase, OafA
A,C	Promoter	cds4042	WP_001541267.1	Hypothetical protein
A,C	Promoter	cds883	WP_000402561.1	Glyoxylate/hydroxypyruvate reductase A, GhrA
A,C	Promoter	cds885	WP_000753742.1	Transposase
B,C	CDS	cds1121	WP_000854400.1	PTS fructose transporter subunit EIIBC
B,C	Promo. & CDS	cds1203	WP_001253818.1	Two-component sensor histidine kinase, EnvZ
B,C	CDS	cds1204	WP_001157751.1	DNA-binding response regulator, OmpR
B,C	Promoter	cds1205	WP_000856695.1	Transcription elongation factor, GreB
B,C	CDS	cds135	WP_000762406.1	Catabolite repressor/activator, FruR
B,C	CDS	cds1840	WP_000193432.1	Two-component system response regulator, RssB
B,C	Promoter	cds1841	WP_000729450.1	UTP-glucose-1-phosphate uridylyltransferase
B,C	Promoter	cds2285	WP_000922811.1	Arginine ABC transporter substrate-binding protein
B,C	CDS	cds677	WP_001046438.1	Outer membrane protein, LpxR


*Lines A–C were samples taken from the ceftiofur tolerant population (>2.0 μg/ml) and grown in parallel for DNA sequencing. Non-synonymous coding region SNPs (CDS), upstream regulatory region SNPs (promoter), both (promo. & CDS). Synonymous coding region SNPs [CDS (syn)].*

The observed genetic changes in the regulatory/promoter regions of the arginine and galactose ABC transporters substrate-binding proteins, aromatic amino acid exporter, CirA drug transporter/catecholate siderophore receptor, heme exporter proteins CcmB, and sugar translocase, and the coding sequence changes in the heme exporter proteins CcmA, sulfate ABC transporter substrate-binding protein, predicted outer membrane porin (LpxR), and PTS fructose transporter subunit EIIBC may function to decrease ceftiofur concentrations in the periplasm, increase export of ceftiofur from the cells, and/or redirect ceftiofur into the cytosol for enzymatic detoxification (Hu et al., 2008 p. 109; Pi et al., 2012, p. 110; Kelley et al., 2015, p. 16). The conserved deletion in the PTS fructose transporter EIIBC gene removes the original start codon, resulting in an 18 amino acid N-terminal truncation, opening up the pore to better accommodate active export of ceftiofur (Hu et al., 2008, p. 109; Kelley et al., 2015, p. 16; **Supplementary Figure 1**). The conserved deletion in the sulfate ABC transporter occurs within a low quality region of the reference genome, so cannot be definitively characterized for comparison, but implies a slightly less bulky internal channel more accommodating to secretion of bulky substrates like ceftiofur.

CirA is an outer membrane active transporter and receptor protein for siderophores, colicins, and microcins able to transport monomers, dimer, and linear trimers of 2,3-dihydorxybenzoylserine (Pi et al., 2012, p. 110) and potentially electrostatically similar compounds such as ceftiofur or a derivative. The observed SNP may render the CirA promoter more active to provide enhanced ceftiofur export. Further genetic assays are required to fully characterize these transcriptional effects. LpxR is a predicted lipid A deacylase outer membrane porin, genetically associated with phosphofructokinase, and the hydrophobic antibiotic resistance protein Omb. The ceftiofur tolerance associated SNP observed in LpxR produces a R303L amino acid substitution near the extracellular collar of the porin structure (Rutten et al., 2009, p. 111; Kelley et al., 2015, p. 16; **Supplementary Figure 2**), with predicted effects on general transporter substrate affinity, which may reduce diffusion of ceftiofur without radically affecting import of other substrates. No association to the predicted deacylase active site is evident (Rutten et al., 2009, p. 111). The modification to the LpxR gene may contribute to reduced downstream expression of phosphofructokinase leading to the decreases abundance we observed as discussed above. Further work is required to assess the extent of transcriptional linkage between the two genes and if the SNP alters transcriptional kinetics.

Wild-type heme exporter proteins CcmA and CcmB facilitate export of heme into the periplasm for cytochrome biosynthesis (Christensen et al., 2007, p. 112). The co-modification of the coding sequence of the CcmH subunit of the hemelyase complex suggests a refinement of the heme transfer machinery to either sequester or inactivate ceftiofur. In the ceftiofur tolerance lineages CcmH shows two amino acid substitutions, G330D and A332T, in the C-terminus of the protein binding NfrG-like domain near the canonical protein binding cleft (**Supplementary Figure 3**). CcmA in these lineages also show two amino acid substitutions, Y8H and E25D, in the N-terminus of the ABC transporter-like region at sites predicted to have mild effects on substrate specificity. CcmB in these resistant lineages show SNPs within the predicted regulatory region.

The UTP-glucose-1-phosphate uridylyltransferase, in combination with the GalF subunit, forms an enzyme that catalyzes the formation of UDP-glucose from UTP and alpha-D-glucose 1-phosphate, regulating cellular levels of UDP-glucose, which if converted to UDP-galactose by UDP-glucose 4-epimerase, feeds into LPS biosynthesis contributing to cell wall stability. The SNP in this gene’s promoter region may alter the regulation of this ORF to better accommodate ceftiofur-related cell wall stress. OafA, sugar translocase, and bactoprenolglucosyl transferase contribute to lipopolysaccharide biosynthesis in *Salmonella*. Modification of the regulation of these enzymes may alter cell wall biosynthesis to counteract the peptidoglycan cross-linking deficiency. These genes are under the control of phase variation regulatory pathways similar to the flagellin protein which showed increased abundance as discussed above.

In high osmolarity, the wild-type EnvZ phosphorylates itself and OmpR, activating a transcriptional response enhancing double membrane/cell wall stability against osmotic stress through up regulation of OmpF and OmpC (Yoon et al., 2009). Changes in OmpR have also been connected with resistance to the antibiotic microcin, and reduced virulence (Yoon et al., 2009). The modified forms of EnvZ and OmpR, observed in our ceftiofur tolerant lines, likely act in similar ways to counter the cell wall destabilizing effects of ceftiofur without radically altering protein function, and may contribute to the more generalized antibiotic tolerance observed. The ceftiofur-resistant EnvZ allele shows a five residue deletion, 28-VTTYL-32, at the periplasmic end of a transmembrane segment of the osmolarity sensor domain. This alters the periplasmic orientation and sensor conformation of that domain to enhance sensitivity to ceftiofur-induced stress as an activator of the cell osmotolerance response as a component of tolerance to ceftiofur. OmpR in the ceftiofur-resistant lineages shows a conserved single amino acid substitution (S132L), in the region between the signal receiver domain (8–120) and the DNA-binding effector domain (143–232) (**Supplementary Figure 4**). This substitution is predicted to alter the hydrophobicity of this intermediate linker region, indirectly altering regulation and solubility (Yoon et al., 2009). Similar tolerance-associated differences exist in homologs of these proteins when compared between environmental isolates with natural susceptibility or tolerance to ceftiofur.

The two-component system response regulator RssB represses generalized stress responses in healthy cells, by promoting proteolysis of the stress factor RpoS (Becker et al., 1999, p. 113). The SNP in RssB produces a R315L substitution facing inward in the C-terminal RpoS regulator domain providing more effective response to the chronic stress of growing with ceftiofur. YjiK is a predicted outer membrane protein exhibiting two conserved amino acid substitutions in the resistant lines, D142N occurring on the outer surface and N147V occurring near, but not within, the active site (**Supplementary Figure 4**). The precise function of wild-type or modified YjiK remains unclear, but is linked to quorum sensing through predicted interaction with SdiA (pfam06977). The substitutions may enhance cell envelop stability or alter quorum sensing to reduce sensitivity to ceftiofur. These changes may also relate to the L-PSP enamine/imine deaminase discussed above which is also involved in quorum sensing (Lambrecht et al., 2012). FruR-like factor, GreB, TtcA, EF-Tu, and the identified helix-turn-helix transcriptional regulator mediate and regulate transcription, the mutations of which in ceftiofur-resistant lines alter the transcription profile as noted throughout this study. Wild-type FruR recognized fructose and similar sugars, inducing fructose utilization pathways and repressing the use of alternative carbon sources (Yoon et al., 2009). FruR deletion reduces virulence in mice (Yoon et al., 2009). The ceftiofur tolerance associated deletion in FruR introduces an early stop codon at residue 42, altering or deleting the majority of the regulator (309 out of 334 amino acids). Homologs to this gene also show differences between susceptible and tolerant environmental isolates.

The gene products of the two *Salmonella* EF-Tu genes (a: AHR65859.2 and b: WP_016740776.1) mediate protein biosynthesis in conjunction with EF-Ts and other factors. Two of the three ceftiofur tolerance associated lineages exhibit a conserved D71H substitution in EF-Tu(a), with sub-populations conserving I61S or H67I substitutions. These sites are associated with conformational response to EF-Ts and GTP binding, suggesting EF-Tu(a) in the tolerant lineages have different regulatory kinetics than the wild-type, potentially contributing to the observed decrease in EF-Ts levels. The EF-Tu(b) gene conserves a number of synonymous SNPs in all three lineages, potentially effecting transcription efficiency of that gene.

Modification to these regulatory proteins in the form of coding SNPs (EnvZ, OmpR, RssB, EF-Tu, and FruR) or regulatory SNPs (EnvZ, helix-turn-helix transcriptional regulator, TtcA, and GreB) alters transcriptional and translational networks, mediating the differential abundance of the proteins discussed earlier (Becker et al., 1999, p. 113; Yoon et al., 2009; Lambrecht et al., 2012). The integrase and transposase regulatory SNPs are likely unrelated to ceftiofur tolerance, instead silencing those enzymes to reduce the potentially deleterious mobilization of prophage and transposons in response to cell stress.

Genetic and regulatory changes in oxaloacetate decarboxylases, formate dehydrogenase-*N* subunit-α, dimethyl sulfoxide reductase, glyoxylate/hydroxypyruvate reductase A, membrane-associated ATP:dephospho-CoA triphosphoribosyl transferase (CitG), the pathogenicity island 2 effector protein (SseI), predicted Ig-like domain repeat molybdopterin-binding oxidase/adhesin, and thiol:disulfide interchange protein may enable interaction with ceftiofur or derivatives as part of uncharacterized detoxification processes. Thiol:disulfide interchange proteins act in the periplasm and cytosol catalyzing formation and breakage of disulfide bonds, control cysteine sulfenylation levels, and rescue oxidatively damaged proteins. Thus, this protein may modify sulfide bonds within ceftiofur or a derivative or chaperon a sensitive cysteine in some other protein involved in ceftiofur tolerance. The conserved regulatory region polymorphisms likely adjust expression to respond to ceftiofur, while the observed K84N substitution in the α-helical anti-reduction domain likely enhances activity at the expense of specificity.

Glyoxylate/hydroxypyruvate reductase A catalyzes the formation of glycolate and glycerate from glyoxylate and hydroxypyruvate, respectively, through reduction of aldehyde or keto groups. This enzyme may catalyze similar reduction of ceftiofur’s thioester, amides, or a derivative under the influence of the observed regulatory SNPs. CitG is a membrane-associated protein which generates 2′-(5″-triphosphoribosyl)-3′-dephospho-CoA as an essential cofactor for malonate decarboxylase. This reaction involves the triphosphoribosylation of an exposed hydroxyl group on the ribose in 3′-dephospho-CoA. While no exposed hydroxyl groups are present in ceftiofur, one or more may be present in intermediate derivatives during detoxification, such as hydroxyl-1,3-thiazine-5-methyl-mercaptan. The altered regulation afforded by the observed SNPs in the CitG gene may thus indirectly contribute to detoxification.

The pathogenicity island 2 effector protein (SseI) in ceftiofur tolerant lineages encodes changes in the upstream regulatory/promoter region of this gene, and a T13I substitution in the N-terminal SGNH hydrolase domain. The precise structural localization of this substitution cannot be definitively predicted due to the limits of modeling confidence. SGNH hydrolases are known for hydrolyzing very diverse substrates (esters, thioesters, amides, lipids, carbohydrates, etc.) with highly flexible induced fit mechanisms (Akoh et al., 2004), thus interaction with ceftiofur may be enabled or enhanced by this substitution. If bound, ceftiofur could be degraded as a thioesterase hydrolysis (**Figure 2g**), similar to ceftiofur degradation in mammals (Beconi-Barker et al., 1995; Li et al., 2011; Wagner et al., 2011), or amide hydrolysis (**Figure 2a**), or co-secreted with SseI via the type III secretion systems.

The ceftiofur-resistant lineages also share an R2364H substitution in the inner membrane, predicted molybdopterin coordinating oxidase/adhesin (cds2374). This gene sequence is 99.5% identical over 12,276 nt to the Ig-like domain repeat protein gene from *S. enterica* Enteritidis SA20094383 (AHQ19329.1) differing in the presence of a frameshift in the C-terminus of the parental ABB07-SB3071 non-resistant strain. Thus, the SA20094383 gene was used to model the unsequencible N-terminal section of the ABB07-SB3071 alleles. The substitution site occurs within or just ahead of the N-terminus of the 19th Ig-like domain repeat near the middle of the protein, 254 residues from the third molybdopterin binding domain and 93 residues from the fourth molybdopterin binding domain. Due to the size and repetitive nature of this protein, precise structural prediction was not feasible. Domain fit modeling places the substitution on the N-terminal loop between Ig-like domains where it may enhance the stabilizing effects of the adhesion function. The predicted molybdopterin coordinating oxidase/reductase activity may also play a role in detoxifying ceftiofur. This family of oxidases/reductases catalyze the formation or breakage of a double bond between an oxygen atom and a substrate with an exposed pair of electrons (e.g., sulfite ↔ sulfate, nitrate ↔ nitrite). The sulfurs in the thioester, thiazine, and thiazole groups, and the nitrogens in the iminomethoxy/ketoxime and thiazole groups in ceftiofur exhibit such oxidizable/reducible electron pairs (**Figure 2**) depending on the specific activity of this protein. Similar reactions with ceftiofur have been demonstrated *in vitro* (Lim et al., 2011). Modifications of the ketoxime group that exposed the β-lactam ring to attack would enhance the detoxifying activities of basally expressed β-lactamases without increasing levels of β-lactamase protein. Coordinated molybdenum has also been found to catalyze the conversion of amides to amines in non-enzymatic contexts (Ugarte et al., 2011), so might reduce the carbon–oxygen double bonds of the thioester or two amides in ceftiofur. Further this gene is encoded 40 nucleotides upstream of the annotated antibiotic ABC transporter ATP-binding protein (WP_000358566.1) potentially altering its expression from polycistronic co-transcripts.

Wild-type oxaloacetate decarboxylases catalyze the decarboxylation of oxaloacetate to form pyruvate and carbon dioxide (Schmid et al., 2002). In *Salmonella*, this reaction occurs in the periplasm through a trimeric integral membrane complex, coupled to sodium translocation by the gamma subunit (Schmid et al., 2002), which in the ceftiofur-resistant lines conserve four SNPs, changing the final three residues from HHV to LNA. Phyre2 could not confidently predict a structural model for this protein preventing precise interpretation of how these substitutions alter the protein function.

An oxaloacetate decarboxylase β-subunit (WP_000444887.1) encodes a six nucleotide insertion (two amino acids, 62-IP-63) into a periplasmic loop in a predicted substrate binding cleft, between two transmembrane domains of the enzyme (**Supplementary Figure 5**). Unrelated ceftiofur tolerant strains also exhibit differences relative to unrelated susceptible strains in this protein. The α-subunit (WP_000150436.1) in the resistant lineages encodes five SNP-derived amino acid substitutions and two inserts in the carboxylase domain (insert 346I, A347P, V348L, L353H, insert 358H, V458L, and A468T), and S542T in the biotin carboxyl carrier protein domain (**Supplementary Figure 6**), potentially modifying the carboxylase activity to extend to ceftiofur or degradation intermediates. All subunits (α, β, and γ) encode SNPs in their predicted promoter region supporting altered regulation kinetics. Decarboxylation is the established second step of detoxification of β-lactam antibiotics (Sauvage et al., 2008). Thus, the SNPs in oxaloacetate decarboxylases may confer altered ion transport, and/or the ability to more efficiently decarboxylate ceftiofur or a derivative. Other oxaloacetate decarboxylase genes showed no change in sequence suggesting this particular set of proteins may be important for ceftiofur tolerance, while the others serve other functions.

Dimethyl sulfoxide reductase catalyzes the conversion of dimethyl sulfoxide to dimethyl sulfide as a reductive dehydration of the sulfoxide group (McEwan et al., 2002). This enzyme may catalyze similar reactions against the oxygens in the thioester, amide, or iminomethoxy/ketoxime groups in ceftiofur (**Figure 2**), or a detoxification intermediate, under the influence of the regulatory and synonymous SNPs in this gene’s coding region. One of the dimethyl sulfoxide reductases conserved in *Salmonella* Enteritidis strains is genetically associated with the gene for PBP 1C (WP_001014765.1), suggesting a possible unrecognized role in cell wall biogenesis and ceftiofur tolerance, as well as sulfur metabolism.

Formate dehydrogenase-*N* is an integral membrane complex catalyzing the conversion of formate to CO_2_ in the periplasm using nitrate as a terminal electron acceptor (Jormakka et al., 2002). The α-subunit, which showed regulatory region SNPs in our assays, is the site of formate oxidation (Jormakka et al., 2002). In the context of ceftiofur, this enzyme may catalyze oxidation of ceftiofur or a derivative at the carbonic acid group potentially as a decarboxylation, or at another neutrophilic site (**Figure 2**). These genetic changes and predicted functional effects are consistent with the observed biotic depletion of free ceftiofur in cultures growing the resistant lineages, as detected by HPLC. There was no variation in the six serotyping loci used in KASP and targeted PCR amplicon sequencing assays for *Salmonella* Enteritidis. This included oxaloacetate decarboxylase genes which did not differ between the ceftiofur tolerant and susceptible lineages.

## Conclusion

Under the stress of ceftiofur concentrations below the established MIC, and in the absence of external sources of novel genetic information, *Salmonella* Enteritidis ABB07-SB3071 accumulates a small number of conserved nucleotide polymorphisms and selectively altered proteomic profiles to adapt existing resources to resist formally bactericidal levels of ceftiofur. The abundances and distributions of select active and passive transporters normally associated with sugar and amino acid metabolism were altered to react to their off target or mutationally facilitated interactions with ceftiofur. As ceftiofur inhibits peptidoglycan cross-linking, these alterations functioned to enhance active drug efflux from the periplasm, decrease passive facilitated diffusion of the drug, and shunt a subset of the drug into the cytoplasm to be detoxified by semi-promiscuous esterases, reductases, and decarboxylases such as pyruvate dehydrogenase and SseI hydrolase. This sequestration in the cytosol is most evident in the 2.0 μg/ml adapted lineage which exhibited 2.9-fold more ceftiofur internally than externally. The enzymatic reactions observed target key structural groups required for inhibition of peptidoglycan cross-linking (β-lactam ring, amino-thiazole) and resistance to β-lactamases (iminomethoxy/ketoxime). This contrasts traditional views in which horizontally transferred β-lactamase are considered a principle cause of resistance to this antibiotic class, rather than repurposed metabolic enzymes. These activities suggest a novel pathway of ceftiofur degradation at work, contributing to the reduction in free ceftiofur present in the resistant compared to the susceptible cultures. Increased binding of ceftiofur to insoluble bacterial components likely also contributes to a significant extent.

As the DIGE assay focused on proteins from the soluble fraction, differential expression of membrane-associated proteins was not directly detectible. Thus, the SNP-based predictions of differential expression of enzymes such as oxaloacetate decarboxylase were outside of the limits of this study. Such compositional changes to the membrane proteins are consistent with the protein abundance and SNPs data, and the observed change in ceftiofur susceptibility.

Further studies on the proteins identified above will elucidate the biochemical mechanisms of detoxification and exclusion of ceftiofur and related antibiotics independent of β-lactamase, or PBP-dependent tolerance mechanisms. These findings indicate unrecognized potential for tolerance adaptations without depending on external sources. Similar studies examining *de novo* induced tolerance within closed genetic systems will be a powerful approach to understanding the development of tolerance in the low complexity pathogen populations selectively enriched in food storage systems, hospital acquired infection, and other human engineered semi-sterile environments.

## Author Contributions

DR performed the computational analysis of the 2D-DIGE with assistance from PS and wrote the manuscript with input from all coauthors. PS carried out the 2D-DIGE assays. Whole genome sequencing and analysis was performed by DL, with metabolic and functional interpretation by DR. SB and MD contributed to experimental design for all assays. DR and MH together performed the HPLC assays. MR performed the KASP and targeted PCR assays, and Sensititre assay. SB was the principle investigator, provided overall guidance, mentorship, and resources throughout the scope of this project.

## Conflict of Interest Statement

The authors declare that the research was conducted in the absence of any commercial or financial relationships that could be construed as a potential conflict of interest.

## Supplementary Material

FIGURE S1 Predicted ribbon model of N-terminally truncated, ceftiofur tolerance PTS fructose transporter. Colored N -> C : Red -> Green. Structure prediction by Phyre2.

FIGURE S2 Predicted ribbon model of ceftiofur tolerance LpxR, with R303L amino acid substitution (purple stick) near the extracellular port of the porin pore. Structure prediction by Phyre2.

FIGURE S3 Predicted ribbon model of ceftiofur tolerance CcmH, with G330D and A332T amino acid substitutions (purple stick) near the protein binding TRP domains (blue), with the interface shown as a space-filling model. The catalytic NrfF domain (red) is unchanged from wild-type. Structure prediction by Phyre2.

FIGURE S4 Predicted ribbon model of ceftiofur tolerance YjiK, with D142N and N147V amino acid substitution (purple stick) near the active site residues (red). Structure prediction by Phyre2.

FIGURE S5 Predicted ribbon model of ceftiofur tolerance oxaloacetate decarboxylase subunit beta, with two amino acid insertion 61-IP-62 (green sticks). Predicted transmembrane domains (blue). Structure prediction by Phyre2.

FIGURE S6 Predicted ribbon model of tolerance oxaloacetate decarboxylase subunit alpha, with two single amino acid insertions 346-I-346 and 358-H-358, and six amino acid substitutions (A347P, V348L, L353H, V458L, A468T, S542T) (purple stick) near the active site residues (red) and carboxyltransferase interaction site (blue). Dimerization domain (green). Structure prediction by Phyre2.

## Abbreviations

CFU: colony forming units
2D-DIGE: two-dimensional fluorescence difference gel electrophoresis
DMF: dimethylformamide
DTT: dithiothreitol
HPLC: high performance liquid chromatography
mAU: milli absorption units
MHB: Müller–Hinton II broth
OD600: optical density at 600 nm
PBPs: penicillin binding transpeptidase proteins
SDS: sodium dodecyl sulfate
