# Supplementary figures and images for: Genomic and Proteomic Analyses of Salmonella enterica Serovar Enteritidis Identifying Mechanisms of Induced de novo Tolerance to Ceftiofur
Source: Front Microbiol. 2018 Sep 10;9:2123. doi: 10.3389/fmicb.2018.02123 (PMC6139387; doi:10.3389/fmicb.2018.02123)

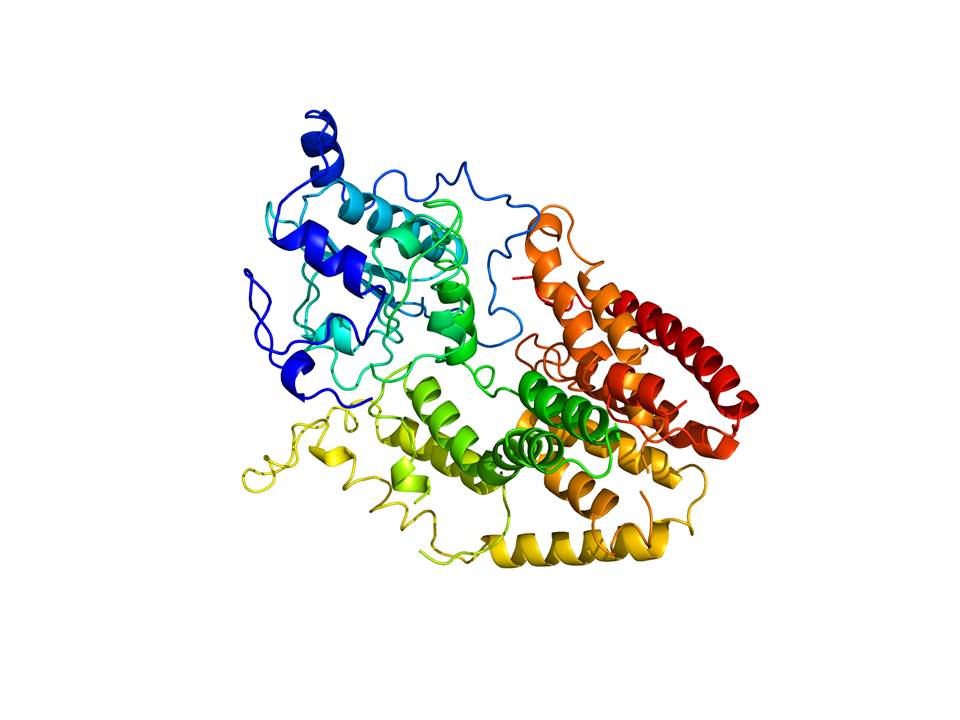

Supplement: FIGURE S1 — Predicted ribbon model of N-terminally truncated, ceftiofur tolerance PTS fructose transporter. Colored N -> C : Red -> Green. Structure prediction by Phyre2. [file Image_1.JPEG]

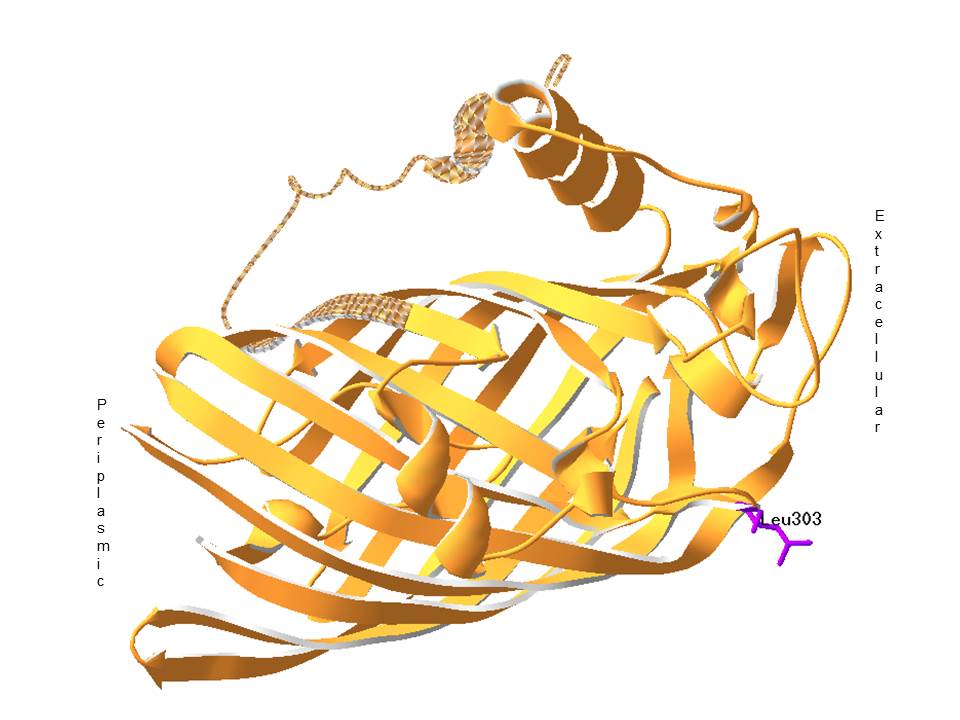

Supplement: FIGURE S2 — Predicted ribbon model of ceftiofur tolerance LpxR, with R303L amino acid substitution (purple stick) near the extracellular port of the porin pore. Structure prediction by Phyre2. [file Image_2.JPEG]

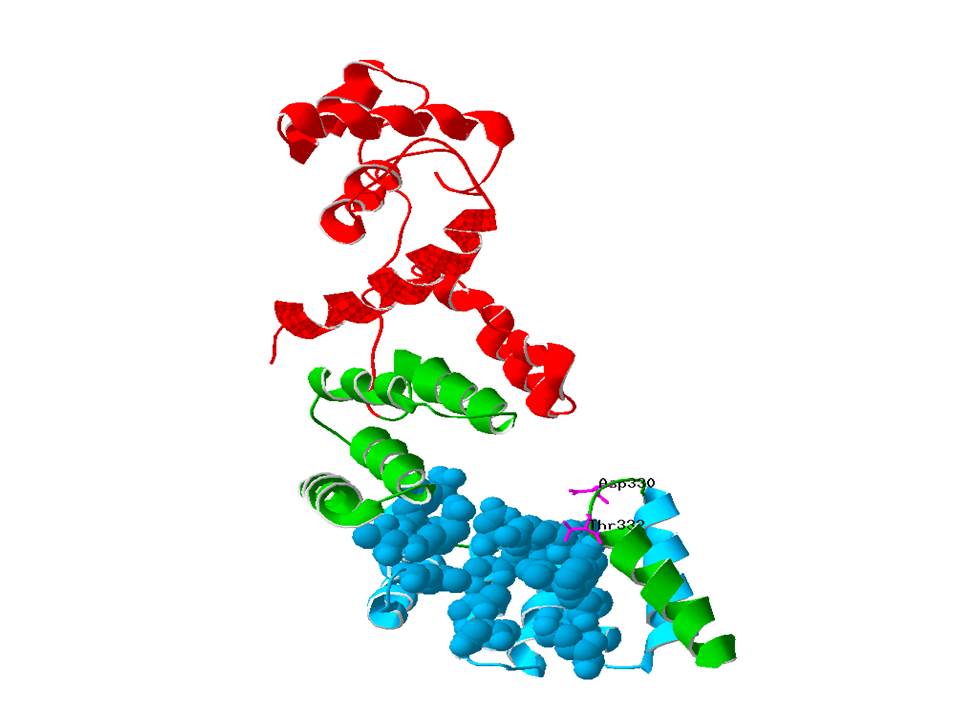

Supplement: FIGURE S3 — Predicted ribbon model of ceftiofur tolerance CcmH, with G330D and A332T amino acid substitutions (purple stick) near the protein binding TRP domains (blue), with the interface shown as a space-filling model. The catalytic NrfF domain (red) is unchanged from wild-type. Structure prediction by Phyre2. [file Image_3.JPEG]

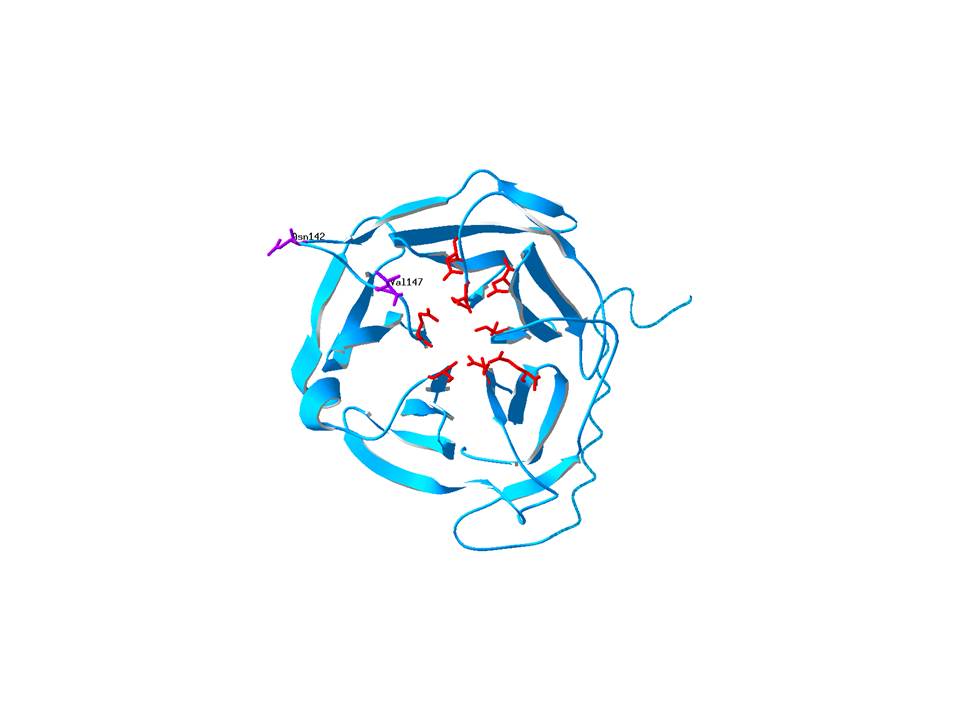

Supplement: FIGURE S4 — Predicted ribbon model of ceftiofur tolerance YjiK, with D142N and N147V amino acid substitution (purple stick) near the active site residues (red). Structure prediction by Phyre2. [file Image_4.JPEG]

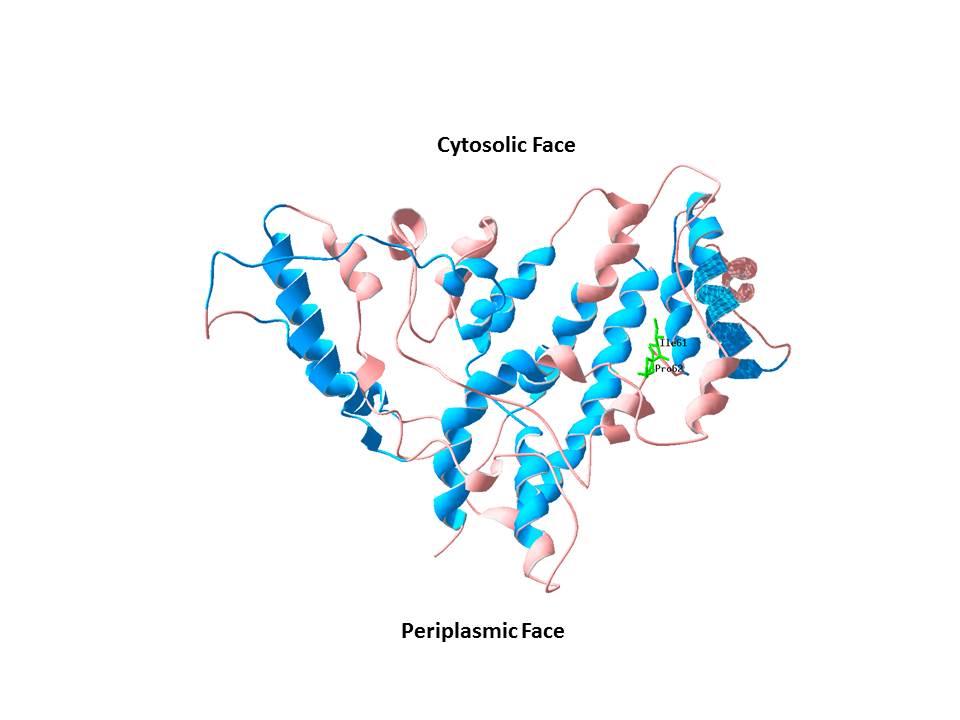

Supplement: FIGURE S5 — Predicted ribbon model of ceftiofur tolerance oxaloacetate decarboxylase subunit beta, with two amino acid insertion 61-IP-62 (green sticks). Predicted transmembrane domains (blue). Structure prediction by Phyre2. [file Image_5.JPEG]

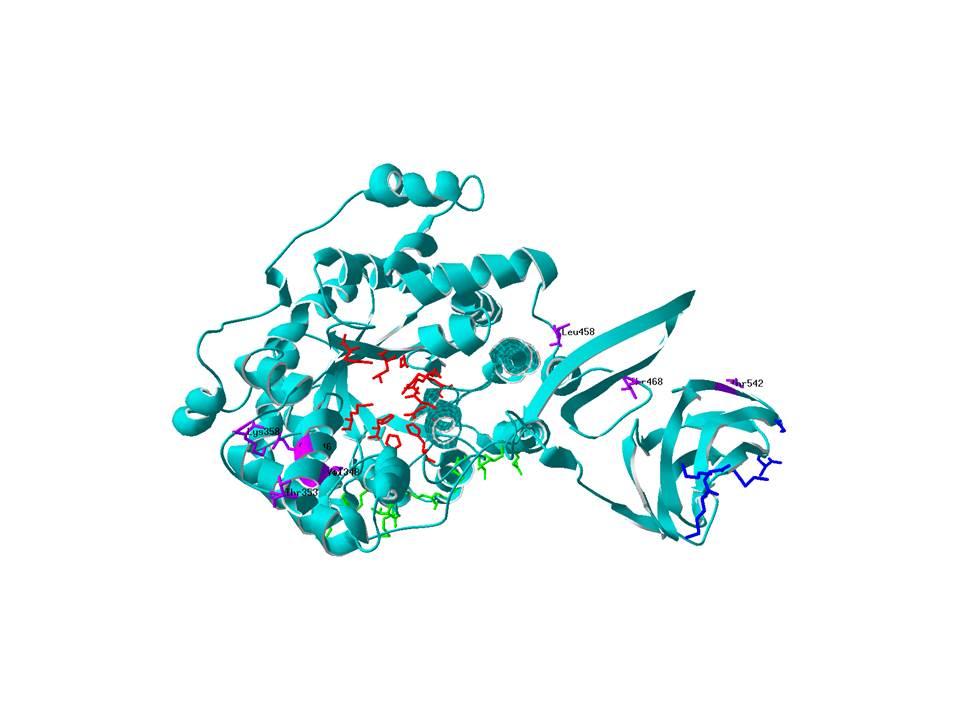

Supplement: FIGURE S6 — Predicted ribbon model of tolerance oxaloacetate decarboxylase subunit alpha, with two single amino acid insertions 346-I-346 and 358-H-358, and six amino acid substitutions (A347P, V348L, L353H, V458L, A468T, S542T) (purple stick) near the active site residues (red) and carboxyltransferase interaction site (blue). Dimerization domain (green). Structure prediction by Phyre2. [file Image_6.JPEG]
